# Two Faces of the Two-Phase Thermodynamic Model

**DOI:** 10.1021/acs.jctc.1c00156

**Published:** 2021-10-14

**Authors:** Ádám Madarász, Andrea Hamza, Dávid Ferenc, Imre Bakó

**Affiliations:** † Research Centre for Natural Sciences, Magyar Tudósok Körútja 2, H-1117 Budapest, Hungary; ‡ Institute of Chemistry, ELTE, Eötvös Loránd University, Pázmány Péter sétány 1/A, H-1117 Budapest, Hungary

## Abstract

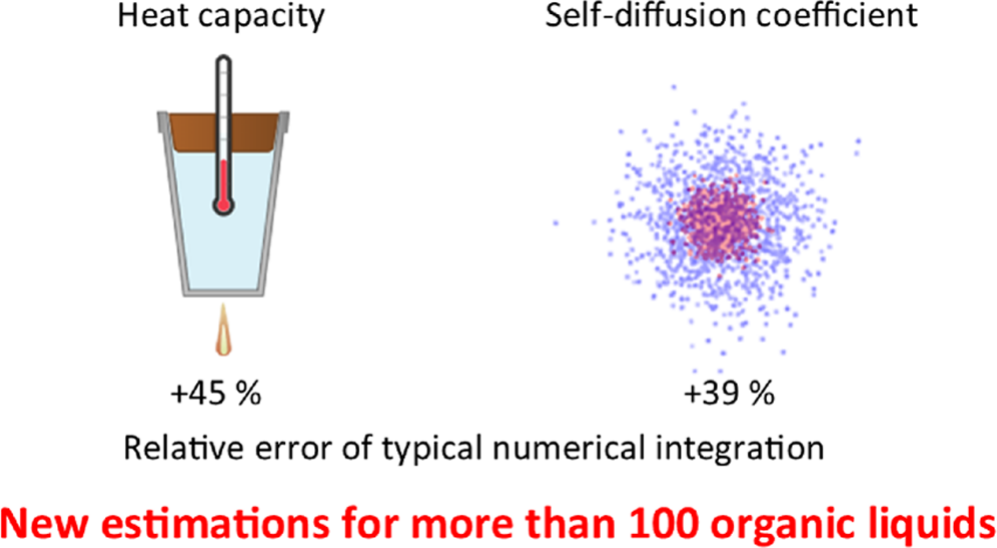

The quantum harmonic model and the two-phase thermodynamic method (2PT) are widely used to obtain quantum-corrected properties such as isobaric heat capacities or molar entropies. 2PT heat capacities were calculated inconsistently in the literature. For water, the classical heat capacity was also considered, but for organic liquids, it was omitted. We reanalyzed the performance of different quantum corrections on the heat capacities of common organic solvents against experimental data. We have pointed out serious flaws in previous 2PT studies. The vibrational density of states was calculated incorrectly causing a 39% relative error in diffusion coefficients and 45% error in the 2PT heat capacities. The wrong conversion of isobaric and isochoric heat capacities also caused about 40% error but in the other direction. We have introduced the concept of anharmonic correction (AC), which is simply the deviation of the classical heat capacity from that of the harmonic oscillator model. This anharmonic contribution is around +30 to 40 J/(mol K) for water depending on the water model and −8 to −10 J/(mol K) for hydrocarbons and halocarbons. AC is unrealistically large, +40 J/(K mol) for alcohols and amines, indicating some deficiency of the OPLS force field. The accuracy of the computations was also assessed with the determination of the self-diffusion coefficients.

## 1 Introduction

Accounting for nuclear quantum effects is essential to obtain meaningful thermodynamic properties that are comparable to experimental observations.^1^ The most typical example is that zero point energies are indispensable for the determination of reaction free energies. The quantum harmonic oscillator model works quite well for small molecules and solid states, but the anharmonicity becomes significant in macromolecules, interfaces, and liquids, and the potential energy surfaces must be mapped using molecular dynamics or Monte Carlo simulation. Berens proposed to add quantum correction to the classically calculated properties using the harmonic oscillator model.^2^ Goddard improved this by the separation of different motions like translation rotations and vibrations and using different partition functions for each of them.^3,4^ This was abbreviated as the two-phase thermodynamic (2PT) model referring to the gas-phase and solid-phase motions in contrast to the one-phase thermodynamic (1PT) method where only vibrations were considered. An anharmonic correction was also included in Berens’ original idea, and thus we refer to that method as one-phase-thermodynamics with anharmonic correction (1PT+AC).

2PT and 1PT+AC methods were successfully applied for the calculation of thermodynamic properties of several systems such as Lennard-Jones fluids,^3,5^ water,^2,4,6−15^ aqueous solutions,^16,17^ molten salts,^18^ organic liquids,^19−21^ carbon dioxide,^22^ urea,^23^ ionic liquids,^24−27^ carbohydrates,^28^ cellulose,^29^ mixtures,^30^ and interfaces.^31−36^ Lately, 2PT was used for the definition of the Frenkel line.^37−40^ Both 1PT/2PT methods are still in continuous development in respect of accuracy and applicability.^41−49^

The 2PT method is the most excellent in the calculation of absolute entropy even from short trajectories. Although the heat capacity is strictly determined from the temperature dependence of entropy according to the laws of thermodynamics, the calculation of the 2PT heat capacity is not as consistent in the literature as the computation of the 2PT entropy. The 2PT abbreviation refers to two conceptually different calculation procedures of the heat capacity in different articles. The classical heat capacities were also taken into account in the calculation of the 2PT heat capacity of water,^9,10,17,33^ but in the case of organic solvents classical heat capacities were discarded.^19−21^ According to refs (19−21) we refer to 1PT and 2PT heat capacities that do not contain anharmonic corrections calculated from classical values. In previous studies, there was no systematic comparison of the effect of this anharmonic correction. In the present study, we fill this gap and analyze the 2PT and 1PT+AC methods in more detail.

Here, we focus on heat capacities to evaluate different types of quantum corrections because they contain a large nuclear quantum effect, and there are accurate experimental data that can be used for the benchmark of force fields.^6,17,19−21,25−27,29,50−58^ In contrast to enthalpy or Gibbs energy, heat capacity is an absolute quantity meaning that there is no need to set the zero point. Additionally, the isobaric heat capacity is a state function, so if we know the *c*_*p*_ as a function of *T* and *p*, the other state functions such as the enthalpy and entropy can be calculated as well. Previously, quantum-corrected thermodynamic properties of organic solvents were investigated in two systematic studies by Pascal and Caleman.^19,20^ For the same solvents, they found similar results: the 2PT heat capacities were in good agreement with the experimental data. Both studies showed that the OPLS force field gave better results than other general force fields such as GAFF or CHARMM. We reanalyzed 113 organic solvents from ref (20) to further test the 1PT+AC and 2PT methods. In total, 21 solvents were omitted from the analysis of heat capacities because their calculated self-diffusion coefficients were under 10^–10^ m^2^/s indicating that these systems do not behave like a real fluid but an amorphous solid.

## 2 Theory

For the determination of the quantum-corrected thermodynamic properties, the velocity autocorrelation functions (VACF) are computed from molecular dynamics simulations that can be defined as follows
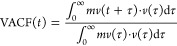
1where *m* is the atomic mass and *v* is the velocity as a function of time (*t*). With this definition, the autocorrelation function is always 1 at zero time, i.e., VACF(0) = 1. The vibrational density of states (VDOS) is the Fourier transform of the autocorrelation function (VACF)
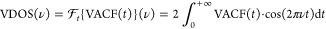
2where *ν* is the frequency.

The Fourier transform of VDOS equals to the VACF
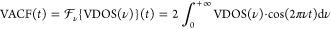
3If we set *t* = 0 in eq 3, then we obtain the norm of VDOS
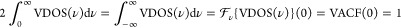
4Originally Berens proposed that the quantum-corrected density of states can be determined by the multiplication of VDOS with an appropriate weight function *w*(2)
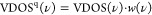
5In the 1PT method there is no separation of motions, and all are considered as vibrations. The quantum weight function for the heat capacity is^59^
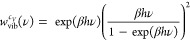
6where β = (*k*_B_*T*)^−1^, *k*_B_ is the Boltzmann constant, *T* is the temperature, and *h* is the Planck constant. Thus, the isochoric heat capacity can be calculated as
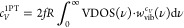
7where *R* is the universal gas constant and *f* = 3*N* is the number of degrees of freedom of an *N*-atomic molecule.

Gaseous motions are separated from vibrations in the 2PT method. The total VDOS is decomposed into two terms, solid and gaseous components
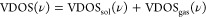
8The gaseous component is determined by VDOS(0) and the fluidity factor *f*_m_
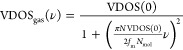
9where *N*_mol_ is the number of molecules. With the definition of eqs 1 and 2, we obtain molar quantities and *N*_mol_ = 1. In an improved version of 2PT, the gaseous and solid components of the VDOS are determined for both translation and rotation.^4^ This decomposition, however, is not really needed because the gaseous component of the rotation is zero as in the case of vibrations. See the derivation in the Supporting Information.

Different weight functions are used for the different motions in the calculation of the 2PT heat capacity^19,20^
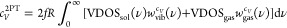
10The weight function of the gaseous component is 1/2 for the heat capacity.

In the 1PT+AC method, a quantum correction (*c*_*V*_^Δ^) is added to the classical isochoric heat capacity (*c*_*V*_^cl^)^2^ as Berens et al. proposed originally^2^
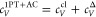
11The quantum correction can be determined from the quantum harmonic weight function. *c*_*V*_^Δ^ given by
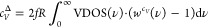
12If the integral terms are partitioned differently, then the 1PT+AC notation becomes apparent
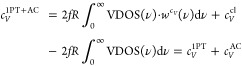
13where the second term is the anharmonic correction
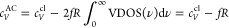
14The 1PT+AC heat capacity is actually a sum of three terms: the heat capacity of *f* classical harmonic oscillators plus an anharmonic and a quantum correction:
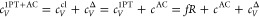
15Jorgensen proposed to correct the classical heat capacity by the estimation of the intramolecular component using the ideal gas value taken from experiments or ab initio calculations.^50,51^ If a given force field reproduces the experimental heat capacity of the gas accurately, then Jorgensen’s approach should give a similar value to the 1PT+AC method. Some deviation may occur if the frequencies of the intramolecular vibrations differ in the liquid and gas phases.

Recently, we have shown that Berens’ original idea about the quantum correction on thermodynamic properties can be extended to structural properties if the quantum correction is applied in the time domain instead of the frequency domain.^60,61^ Our technique, the generalized smoothed trajectory analysis (GSTA) gives identical results for thermodynamic properties as 1PT+AC. For instance, the heat capacity can be obtained from the VACF directly
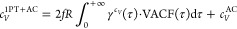
16where *γ*^*c*_*V*_^ is the Fourier transform of the weight function in eq 6
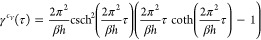
17where csch is the hyperbolic cosecant function. This formalism allows a much more effective calculation because there is no need to calculate the VDOS.

The isobaric heat capacity can be determined from the isochoric heat capacity by employing the relation
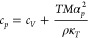
18where α_*p*_ denotes the thermal expansion coefficient, *M* is the relative molar mass, ρ is the density, and *κ*_*T*_ is the isothermal compressibility. The isobaric 1PT+AC heat capacity is computed as a sum of the classical isobaric heat capacity and the quantum correction from eq 15, and the latter can be determined from VACF or VDOS according to eqs 7 and 16
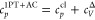
192PT can also be combined with an anharmonic correction that satisfies the correspondence principle
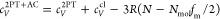
20This definition of the 2PT+AC heat capacity may correspond to previous 2PT calculations in the literature, where the classical heat capacities were also taken into account.^9,10,17,22,33^

## 3 Methods

We performed 10.6 ns long *NpT* simulations to determine the isobaric heat capacities and self-diffusion coefficients using GROMACS simulation software.^62^ The settings and inputs were taken from ref (20) (the input files can be found in the Supporting Information). The cubic box always contained 1000 molecules. A cutoff of 1.1 nm was employed for the intermolecular interactions. The particle mesh Ewald algorithm was used for the computation of the Coulomb interactions. The time constants of the Nose–Hoover thermostat and the Parrinello–Rahman barostat were 1.0 and 5.0 ps, respectively. To determine the classical heat capacity including all vibrations, no constraints were applied on bonds, they remained flexible, and thus a 0.2 fs time step was used. 2PT heat capacities were calculated with the “dos” analysis tool of GROMACS. The classical heat capacity was determined from the fluctuation of enthalpy
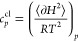
21

## 4 Results and Discussion

### 4.1 Heat Capacity

According to the correspondence principle, the quantum calculations should agree with the classical results as the *h* Planck constant formally approaches zero. The 1PT model gives *fR* for the heat capacity in the classical limit. Applying the classical weight functions of 1 and 1/2 in eq 10, it is easy to see that the 2PT model can give values between *fR*/2 and *fR* for the isochoric heat capacities in the classical limit. The 1PT+AC and 2PT+AC models always satisfy the correspondence principle in contrast with the 1PT or 2PT methods
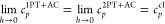
22This also implies that the technique is able to describe the effects of anharmonic motions. The 1PT and 2PT isochoric heat capacities for a rigid water model with 3 translational and 3 rotational degrees of freedom cannot be higher than 6*R* = 49.9 J/(mol K). The fact that in refs (9, 10, 17, 33), the calculated heat capacities are in the range of 57 and 81 J/(K mol), which is significantly larger than the theoretical limit of 49.9 J/(K mol), implies that the anharmonic contribution was also considered. Since the experimental isochoric heat capacity is 74.5 J/(K mol), the anharmonic contribution is at least 24.6 J/(K mol).^63^ From previous simulations, the calculated anharmonic correction is around 30–40 J/(K mol) depending on the water model.^60^

**Figure 1:**
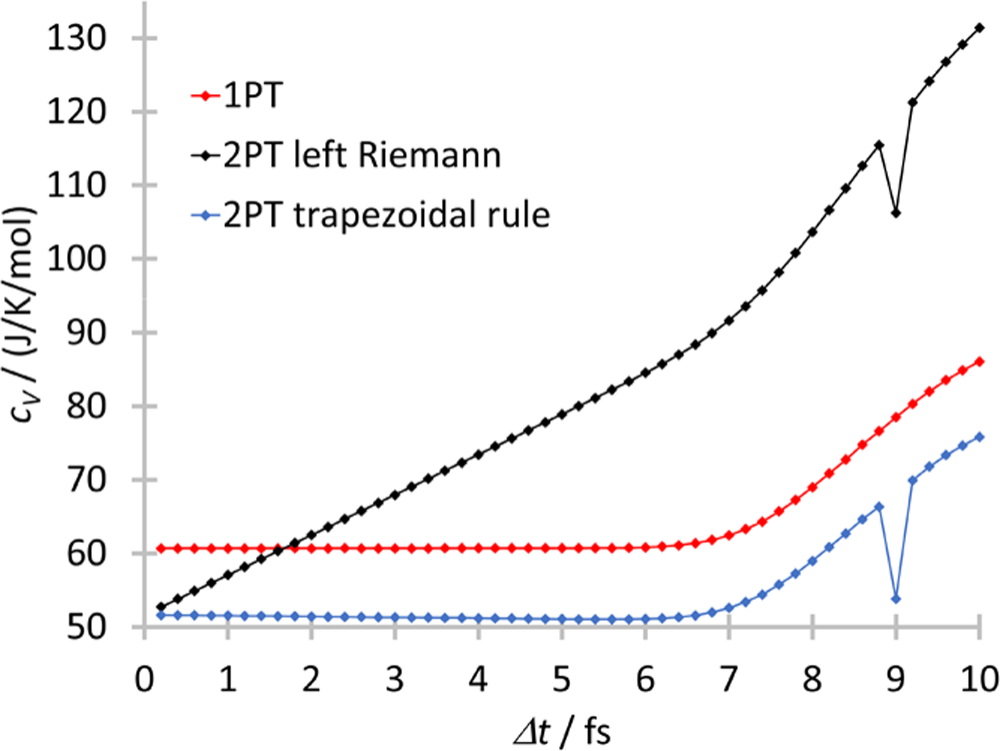
Convergence of the isochoric heat capacity of methanol as a function of time interval.

In our previous study, we showed that the 1PT+AC heat capacity can be significantly overestimated if the left Riemann sum is used instead of the trapezoidal rule in the computation of VDOS in eq 2.^60^ To check whether this numerical error can occur in the calculation of the 2PT heat capacities, we thoroughly tested the “dos” analysis tool of GROMACS that was used in ref (20). The default fast Fourier transformation routine in GROMACS applies the left Riemann rule, but we also implemented a simple trapezoidal integral rule. We analyzed the effect of the different algorithms on methanol. Decreasing the time interval of the integration, the trapezoidal integral converged rapidly at 5 fs, meanwhile the default left Riemann sum gave the correct 2PT heat capacity, 51.6 J/(K mol) only in the Δ*t* → 0 limit (see Figure 1). At a 4 fs time interval, which is generally used in 2PT calculations, the heat capacity is 73.4 J/(K mol) which means a 40% overestimation of the correct value. This agrees well with the result of 75.8 J/(K mol) from ref (20). There is a breaking point for the 2PT heat capacities at 9 fs. This is due to the fact that the period of OH vibrations is exactly 9.0 fs, and this coincidence causes a large uncertainty in the calculation of VDOS at zero frequency. The convergence of the 1PT method is also shown with the trapezoidal formula, and at 4 fs the 1PT heat capacity is also converged with 60.7 J/(K mol).

When we recalculated the heat capacities of nine common solvents from refs (19) and (20) with both integral formulas, we reproduced the literature data but we obtained 45% lower heat capacities with the correct integral formula (see the Supporting Information). In refs (19) and (20) the 2PT heat capacities are similar to each other for the same solvents with the OPLS force field, which implies that in both works, the same (incorrect) integration routine was used.

Surprisingly, in these previous studies excellent correlations were found between the 2PT and experimental isobaric heat capacities. How is it possible that such a good correlation has been achieved, if the values were overestimated by 45%? It seems that there was an (un)fortunate error cancellation, where the opposite error is connected to the conversion between the isobaric and isochoric heat capacities in eq 18.

In ref (20) the *c*_*p*_ – *c*_*V*_ difference is always smaller than 0.1 J/(K mol), and in ref (19) this correction was not larger than 1.2 J/(K mol). We recalculated the *c*_*p*_ – *c*_*V*_ differences for the organic liquids from ref (20) and we obtained orders of magnitude higher values. In our computations, the average difference is 38.4 J/(K mol) and the heat capacity ratio is 1.31. For a few molecules, there are direct experimental data for the isochoric heat capacities (see the Supporting Information). For instance, the *c*_*p*_ – *c*_*V*_ differences of methanol and ethanol are 14 and 11 J/(K mol), respectively.^64,65^ This supports the fact that we calculated the *c*_*p*_ – *c*_*V*_ values correctly.

We also tried to reproduce the heat capacity of methanol from ref (19) using the same simulation software, LAMMPS and the same program code that determined the heat capacities. Although we obtained the same total VDOS with LAMMPS as we did with GROMACS, the solid part was quite different (see the Supporting Information). It turned out that the solid part of the VDOS function is calculated incorrectly in GROMACS because the number of atoms was used instead of the number of molecules in eq 8. In Pascal’s current 2PT code, the FFT algorithms give the numerically exact results and the classical heat capacity is also taken into account in the final heat capacity.

We also determined the 2PT+AC heat capacities according to eq 20. The 2PT+AC heat capacities are always slightly higher than the 1PT+AC heat capacities, and the maximum difference is 0.33 J/(K mol), which is definitely smaller than the uncertainty of the calculations. Since the 2PT+AC heat capacities are almost identical with the 1PT+AC heat capacities (see the Supporting Information), we discuss only the 1PT+AC heat capacities in the rest of the paper.

**Figure 2:**
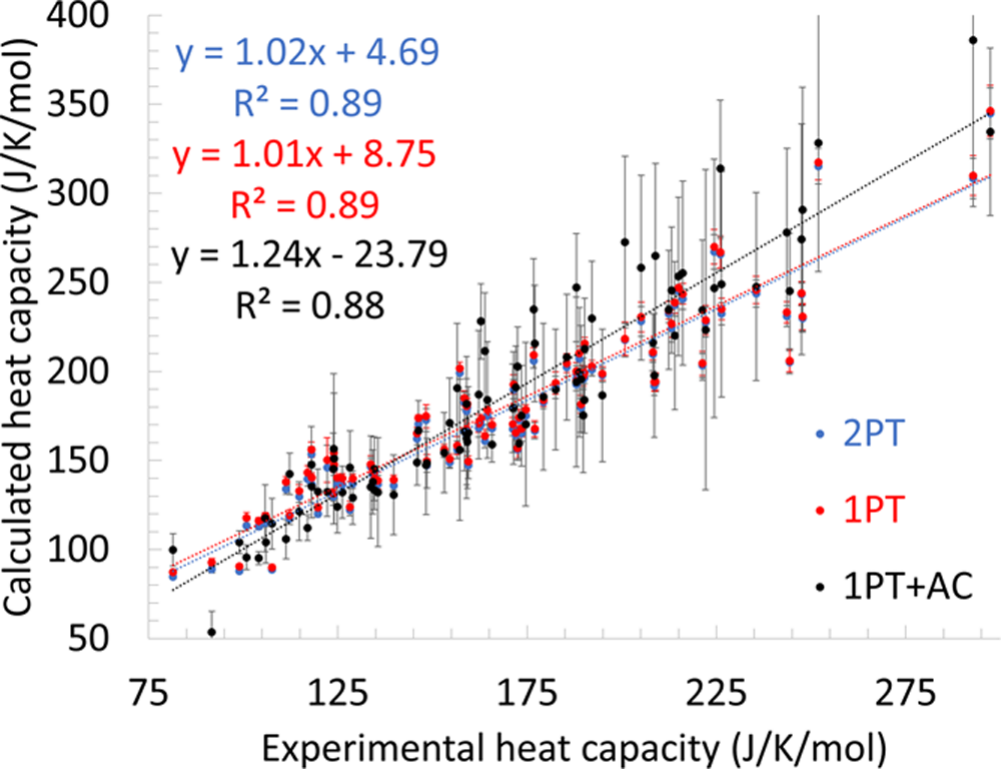
Calculated vs experimental isobaric heat capacities.

The correctly calculated isobaric heat capacities are shown in Figure 2 as a function of experimental values. The overall correlations are good for the predicted and experimental heat capacities, and the *R*^2^ is about 0.9 for all three methods. From the fitted lines it can be seen that the slope of the 1PT and 2PT are almost perfect 1.01 and 1.02, respectively, but for 1PT+AC the slope is 1.24. Both 1PT and 2PT overestimate the isobaric heat capacities. The 2PT model yields systematically lower heat capacities than 1PT with around 2.1 J/(K mol), which can be easily explained by the fact that 2PT considers gaseous motions as well that have a smaller heat capacity than vibrations (*R*/2 vs *R* per degree of freedom). The methods perform differently for different types of compounds, and even their relative goodness is varying.

**Figure 3:**
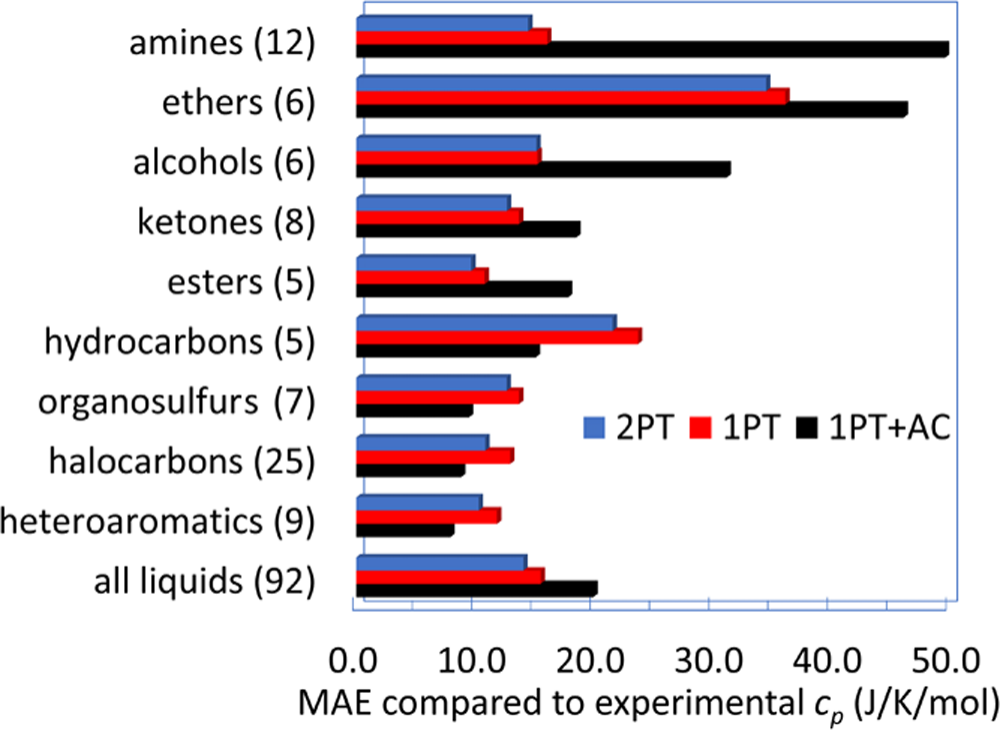
Mean absolute errors (MAE) of the calculated isobaric heat capacities compared to the experimental data.

The mean absolute deviations for different types of molecules are shown in Figure 3. For all of the compounds, the error is 20 J/(K mol) for the 1PT+AC method. The error is smaller with 1PT and even smaller with the 2PT method. For hydrocarbons, organosulfurs, halocarbons, and heteroaromatics the 1PT performs the worst, and the 1PT+AC and 2PT performs similarly better. For amines, ethers, alcohols, and ketones the 1PT+AC performs the worst, and the 1PT and 2PT methods perform much better. The large errors of the 1PT+AC heat capacities may originate from the deficiency of the force field and/or from the inaccuracy of the 1PT+AC method. To separate these two errors, we investigated the classical limits that characterizes the failure of the method.

**Figure 4:**
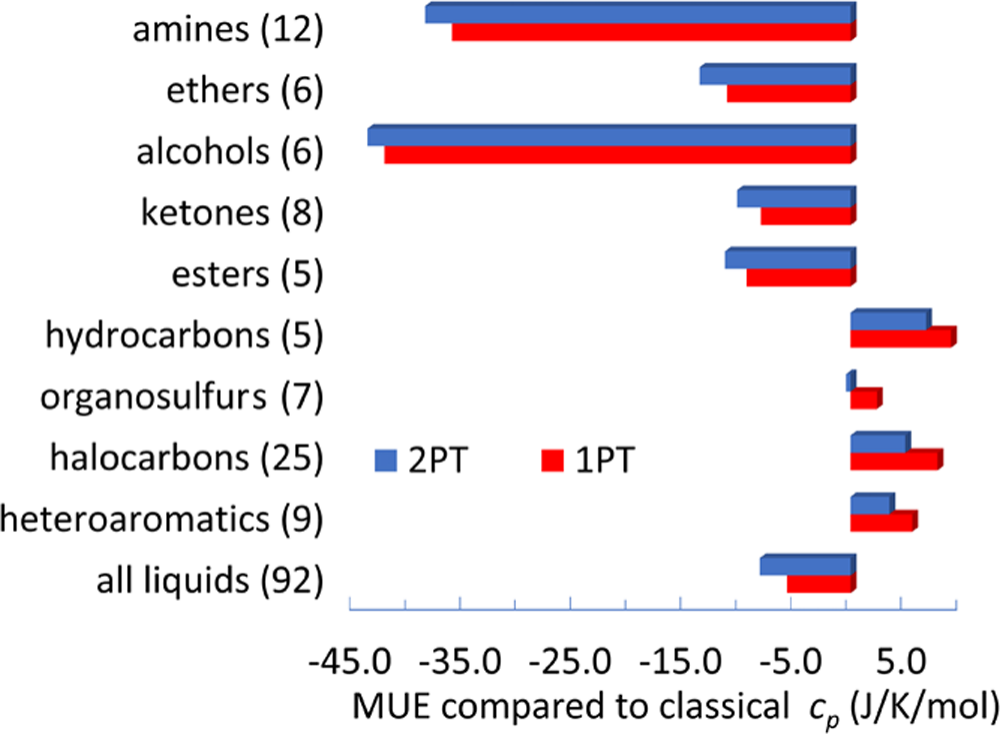
Mean unsigned errors (MUE) of the isobaric heat capacities compared to the classical limit.

As mentioned above, the 1PT and 2PT models do not satisfy the correspondence principle, and they cannot reproduce the classical heat capacities of anharmonic cases. To quantify these deviations, the mean error of the reproduction of the classical heat capacities is shown for the 1PT and 2PT methods in Figure 4. 1PT+AC and 2PT+AC are not shown because their errors are zero, according to eq 22. This kind of error of 1PT equals the negative of the anharmonic correction in the 1PT+AC model. 2PT always underestimates the classical heat capacity with 8.2 J/(mol K) in average, which is comparable to the mean absolute error relative to the experiments, 14.2 J/(mol K). The 1PT method overestimates the classical heat capacity for the heteroaromatics, halocarbons, organosulfurs, and hydrocarbons and underestimates for the other compound groups. This classification correlates perfectly with the relative performance of 1PT and 1PT+AC in Figure 3. If the 1PT heat capacity agrees better with the experiment than the 1PT+AC, then it means that the anharmonicity is described incorrectly by the force field. The Lennard-Jones potential is known to be too repulsive and this may cause inaccuracies when stronger attractive interactions are also present. The uncertainty of the anharmonicity is too large with the OPLS force field for the aliphatic N and O compounds, and this is why the 2PT method estimates the heat capacity of organic liquids more accurately. These results suggest that the effect of anharmonicity is significantly smaller than the quantum effect on the heat capacity of the organic liquids. In the 1PT+AC method, this means that the magnitude of the anharmonic correction is smaller than that of the quantum correction in eq 15.

Caleman and Pascal concluded from their studies that the reproduction of the experimental heat capacities could be improved by a better description of the force constants of bonds and angles.^19,20^ This is true for the quantum correction, but the anharmonic correction can be adjusted with the nonbonding parameters. Our results indicate that the thermodynamic properties are more sensitive to the intermolecular interactions than to the intramolecular interactions. This is in line with the general experiences that in the simulation of the organic liquids, the bond lengths and angles can be constrained at room temperature.

### 4.2 Self-Diffusion

**Figure 5:**
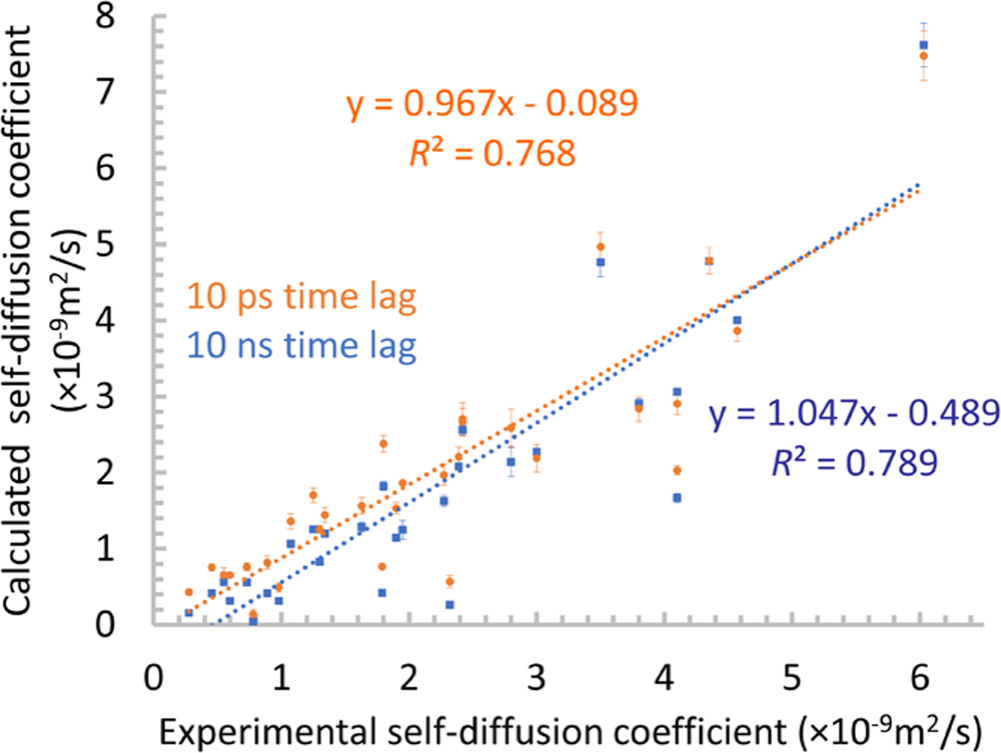
Calculated vs experimental self-diffusion coefficients for 31 organic solvents.

To estimate the consistency of the calculations of VACF and VDOS functions, we computed the self-diffusion coefficients with two different methods.^66^ First, we determined *D*_s_ from the VACF
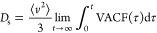
23The self-diffusion coefficient can also be calculated from the mean-squared displacement of the atoms using the Einstein equation
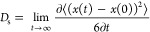
24We computed the self-diffusion coefficients according to these equations with a time lag of 10 ps. The two complementary approaches gave almost identical results (MAE = 0.05 × 10^–9^ m^2^ s^–1^, *R*^2^ = 0.998), which validates how we calculated the VACF and VDOS functions (see the Supporting Information). If we use the less accurate left Riemann sum in eq 23 with the time interval of 4 fs, then the self-diffusion coefficients are overestimated by 39 %. The self-diffusion coefficients were also determined from the 10 ns simulations using eq 24. Compared to the available experimental data, the mean absolute error is 0.62 × 10^–9^ m^2^ s^–1^ for 31 liquids, which means significant correlation (*R*^2^ = 0.79) (see Figure 5). In most cases the differences between the 10 ps and 10 ns values are larger than the uncertainty of the self-diffusion coefficients. Previous studies already showed that a 10 ps time lag is not long enough for converged self-diffusion coefficients (see in ref (67) and further references therein.) The application of a 10 ns time lag instead of 10 ps, however, does not improve the accuracy of the self-diffusion coefficient compared to the experiment. Actually, the mean absolute error is slightly lower, 0.55 × 10^–9^ m^2^ s^–1^ using 10 ps long VACF functions, and the correlation coefficient is almost the same (*R*^2^ = 0.77).

### 4.3 Efficiency of Different Algorithms

Computation of the heat capacity from a converged VDOS function is a memory-intensive calculation since all of the velocities need to be read from several picosecond long trajectories with time intervals of a few femtoseconds. This computational cost can be reduced significantly with the GSTA approach. In the present case, if the VACF function is corrected with eq 16, it is enough to compute the VACF 68 fs long, instead of the 10 ps length used for the determination of the VDOS.

The calculation of the self-diffusion coefficient from a trajectory is orders of magnitude more expensive with the Green–Kubo method than with the Einstein equation. While in the first case we needed to compute 10 ps long VACF with a resolution of 4 fs, in the latter case it was enough to calculate the mean-squared displacement at 8 and 12 ps. Moreover, the calculation of VACF is almost unfeasible with a time lag of 100 ps or longer, when the self-diffusion coefficient converges.

## 5 Conclusions

As a summary, we compared the 2PT and 1PT+AC heat capacities. We pointed out that previous 2PT heat capacities in the literature were calculated incorrectly.^19−21^ We think that the correct 2PT heat capacities include the anharmonic correction (denoted as 2PT+AC in the present paper). The right program code is given in the Supporting Information to calculate the correct 2PT, 1PT, and 1PT+AC methods using GROMACS software. Based on our benchmark calculations, we suggest to use different methods for different purposes. Despite the 2PT method not satisfying the correspondence principle, it can give reasonable estimation for thermodynamic properties of organic liquids. If someone wants to benchmark force fields, or develop new force field parameters it is recommended to use the 1PT+AC method, which accounts for the anharmonicity correctly. Our results help to improve the accuracy of the calculated thermodynamic properties of large systems, and with the use of more efficient algorithms even larger systems can be investigated.

## Supporting Information Available

Simulations along with the program codes, input files, and results (ZIP)
