# Supplementary material for: Two Faces of the Two-Phase Thermodynamic Model
Source: J Chem Theory Comput. 2021 Oct 14;17(11):7187–94. doi: 10.1021/acs.jctc.1c00156 (PMC8582254; doi:10.1021/acs.jctc.1c00156)
Supplement: Supplementary file 1 — ct1c00156_si_001.zip [file ct1c00156_si_001.zip › Supporting_Information.pdf]

Supporting Information for *Two Faces of the Two-Phase Thermodynamic model*

Ádám Madarász, Andrea Hamza, Dávid Ferenc, Imre Bakó

Contents

Test of numerical integrals S2
Heat capacity S2
Calculation of the self-diffusion coefficient S2
Gaseous component of rotation and vibration S2
VDOS function of methanol S3
VDOS functions with 9 fs sampling time S5

Test of numerical integrals

We analyzed a 20 ps long trajectory of a methanol simulation. The velocities were saved in every time step of 0.2 fs. 10 ps long VACF was determined. The VDOS and the heat capacities were calculated using different integration steps from 0.2 to 10 fs.

Heat capacity

After 1 ns long equilibration the classical heat capacities were determined from 9x1 ns long simulation. The values are given as the average of nine calculations with its standard deviation.

Quantum corrections were determined from 5 separated 20 ps long simulations. The velocities were saved in every 4 fs time steps. Between two production run 100 ps long simulations were performed to get independent data.

Calculation of the self-diffusion coefficient

We determined the self-diffusion coefficients with the Green-Kubo formula integrating the VACF function from zero to 10 ps from the output of the **dos** analysis tool, and the error is given as a standard deviation of 9 datapoint. To determine the self-diffusion coefficient at 10 ps from the Einstein equation we used the mean squared displacement at the 8 and 12 ps. From the 10 ns long trajectories the self-diffusion coefficient was computed with the **msd** analysis tool of the GROMACS software with linear fitting on the mean squared displacement vs. time function from 1 to 9 ns.

Gaseous component of rotation and vibration

Here we prove that there is no gaseous component of the rotational or vibrational VDOS. Let us investigate *N* molecules, and one molecule consists of *n* atoms. For simplicity we use one dimension. The centre of mass of the *i*-th molecule at the time *t* is:

$$x_i^c(t) = \frac{\sum_j^n x_{i,j}(t)}{n} \tag{S1}$$

The mean squared distance without translation is:

$$MSD_{rv}(t) = \langle \sum_{i,j}^{N,n} \left(\left(x_{i,j}(t) - x_i^c(t) \right) - \left(x_{i,j}(0) - x_i^c(0) \right) \right)^2 \rangle \tag{S2}$$

Eq S2 can be written as the sum of three parts:

$$MSD_{rv}(t) = \sum_{i,j}^{N,n} \langle \left(x_{i,j}(t) - x_i^c(t)\right)^2 \rangle + \langle \left(x_{i,j}(0) - x_i^c(0)\right)^2 \rangle - 2\langle \left(x_{i,j}(t) - x_i^c(t)\right)\left(x_{i,j}(0) - x_i^c(0)\right) \rangle \tag{S3}$$

As the time approaches infinity in an ergodic system, the first two terms become equal and the last term approches zero:

$$\lim_{t\to\infty} MSD_{rv}(t) = 2\sum_{i,j}^{N,n} \langle \left(x_{i,j}(0) - x_i^c(0)\right)^2 \rangle \tag{S4}$$

Eq S4 means that the $\mathrm{MSD}_{\mathrm{rv}}(t)$ function approaches a constant value with increasing time, therefore its derivative becomes 0 at infinite time:

$$D_{rv} = \lim_{t\to\infty} \frac{\partial MSD_{rv}(t)}{2\partial t} = 0 \tag{S5}$$

This indicates that there is no diffusive part of rotation or vibration.

We have to note that rotational diffusion coefficient can be calculated from the Fourier transform of the autocorrelation of the angular velocity, but in that case the sum of the VDOS of the translation, rotation and vibration is not equal to the total VDOS. To illustrate this problem, let us consider a simple rigid rotator with constant angular velocity of ω. In that case the autocorrelation function of the angular velocity is constant, and the corresponding VDOS function is a Dirac delta function at zero frequency. If the angular velocity is constant, then the (linear) velocities change as a sum of a cosine and a sinus function. If we calculate the VACF from (linear) velocities, then the VDOS is a Dirac delta function at $\omega/2\pi$ frequency. Obviously, this frequency should appear in the calculation of the total VDOS, which means that the rotational VDOS should be determined from (linear) velocities.

VDOS function of methanol

We obtained identical VDOS functions from the simulation of methanol with GROMACS and LAMMPS. The VDOS functions were determined with the "dos" utility from GROMACS simulation.[1] Pascal's 2PT code[2] was used for the analysis of the LAMMPS simulations.[3] The total VDOS functions are almost identical in Figure S1, but the gaseous part of the VDOS are different in Figure S2. This difference is due to fact that

[1] http://www.gromacs.org/
[2] https://github.com/atlas-nano/2PT
[3] https://www.lammps.org/

the number of atoms is used in the “dos” utility of GROMACS instead of the number of molecules. After the correction of this mistake, the two functions become similar.

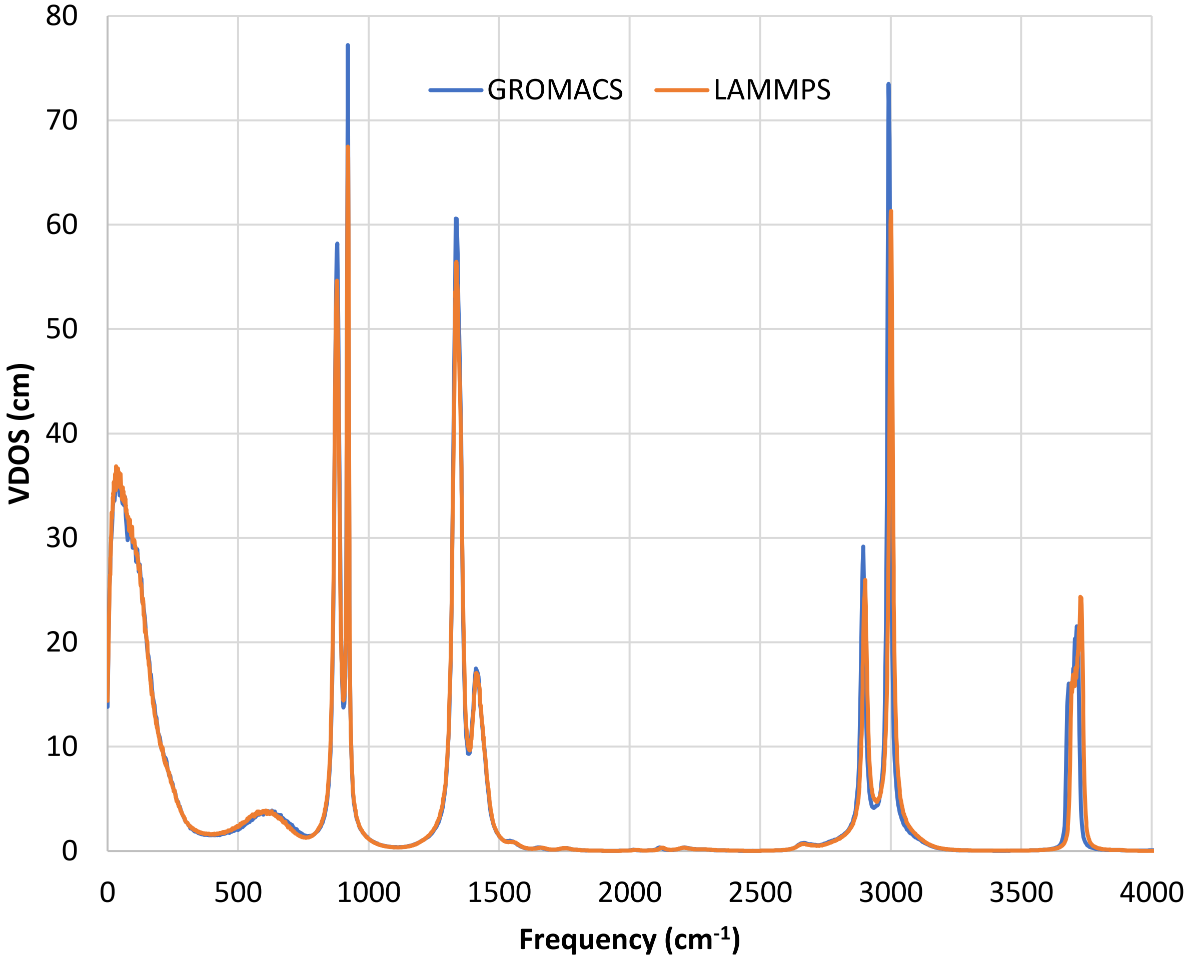

Figure S1. Vibrational density of states of methanol computed with different simulation programs.

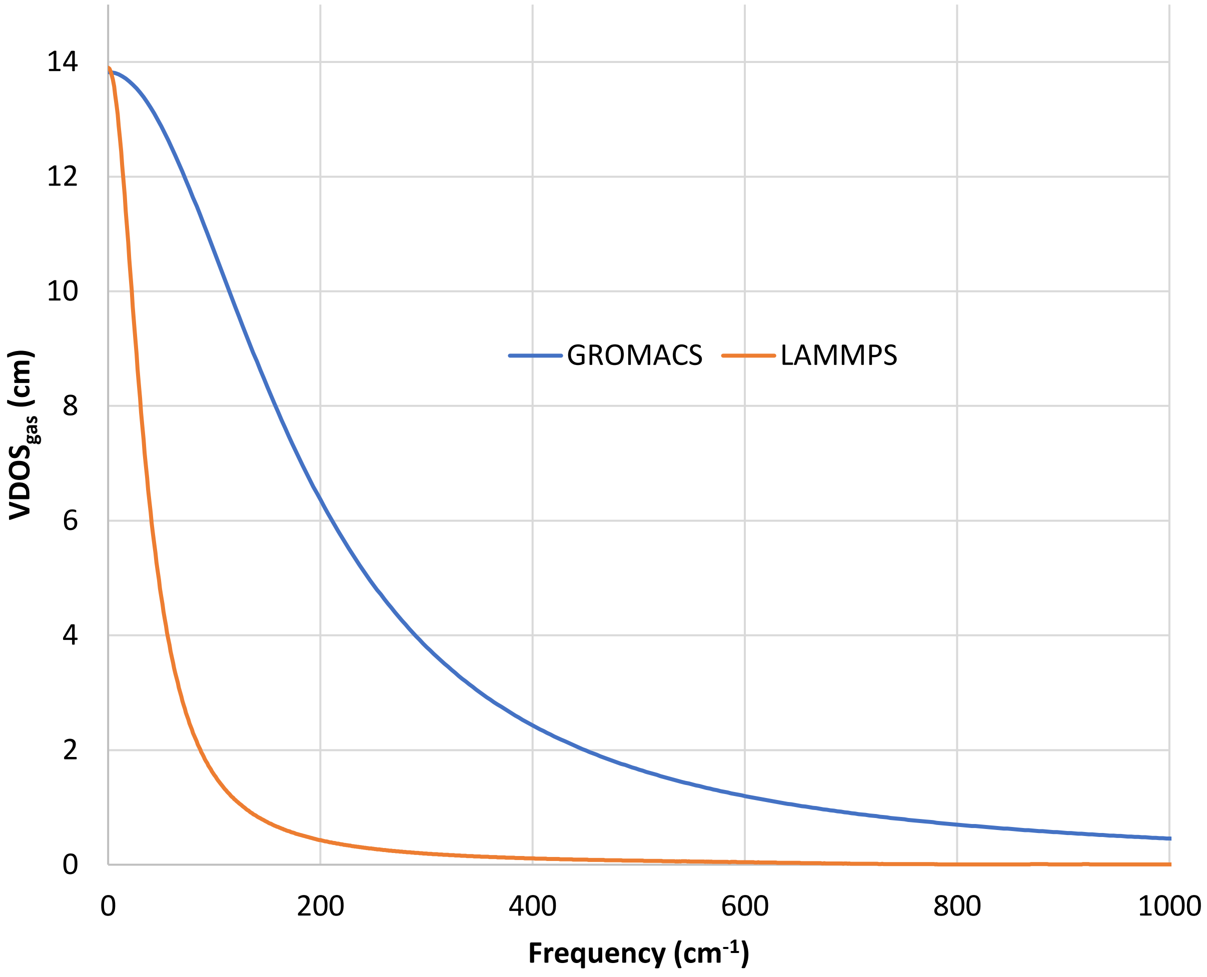

Figure S2. Gaseous part of the vibrational density of states of methanol computed with different simulation programs.

VDOS functions with 9 fs sampling time

There is a breakpoint in the 2PT heat capacities at the time interval of 9 fs in Figure 1, but there is no similar breakpoint for the 1PT heat capacity. This breakpoint is an artifact due to the OH vibration. The low frequency parts of the VDOS functions are shown in Figure S3. The gaseous part of the VDOS function is determined from the total VDOS function at zero frequency in the GROMACS implementation. Using 9 fs interval in the calculation of the VDOS function rises artificially to around 50 cm at zero frequency, while the correct value is 13.9 cm. In Pascal's code (in the LAMMPS implementation), the gaseous part of the VDOS is determined from the VDOS of the center of masses of the molecules, and thus it gives the correct gaseous part of the VDOS function. Since the 1PT heat capacity is not sensitive to the value of the VDOS function at zero frequency, there is no breakpoint in Figure 1 at 9 fs.

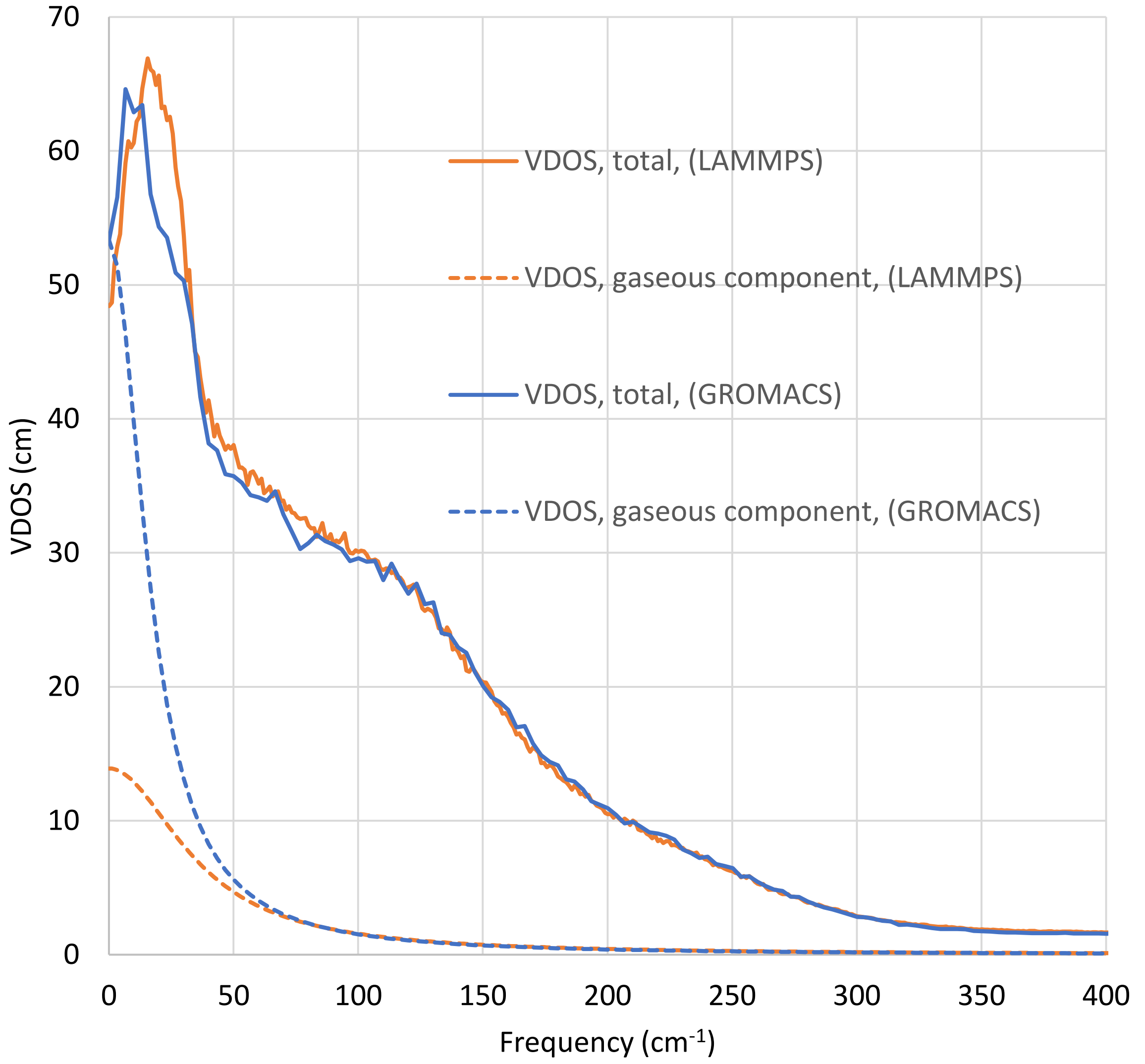

Figure S3. Vibrational density of states of methanol computed with different simulation programs with 9 fs sampling time that corresponds to the period of the OH vibration
